# Exploring the Chemical Space around 8-Mercaptoguanine as a Route to New Inhibitors of the Folate Biosynthesis Enzyme HPPK

**DOI:** 10.1371/journal.pone.0059535

**Published:** 2013-04-02

**Authors:** Sandeep Chhabra, Nicholas Barlow, Olan Dolezal, Meghan K. Hattarki, Janet Newman, Thomas S. Peat, Bim Graham, James D. Swarbrick

**Affiliations:** 1 Medicinal Chemistry, Monash Institute of Pharmaceutical Sciences, Monash University, Parkville, Australia; 2 CSIRO Division of Materials, Science and Engineering, Parkville, Australia; University of Delhi, India

## Abstract

As the second essential enzyme of the folate biosynthetic pathway, the potential antimicrobial target, HPPK (6-hydroxymethyl-7,8-dihydropterin pyrophosphokinase), catalyzes the Mg^2+-^dependant transfer of pyrophosphate from the cofactor (ATP) to the substrate, 6-hydroxymethyl-7,8-dihydropterin. Recently, we showed that 8-mercaptoguanine (8-MG) bound at the substrate site (*K_D_* ∼13 µM), inhibited the *S. aureus* enzyme (*Sa*HPPK) (IC_50_ ∼ 41 µM), and determined the structure of the *Sa*HPPK/8-MG complex. Here we present the synthesis of a series of guanine derivatives, together with their HPPK binding affinities, as determined by SPR and ITC analysis. The binding mode of the most potent was investigated using 2D NMR spectroscopy and X-ray crystallography. The results indicate, firstly, that the SH group of 8-MG makes a significant contribution to the free energy of binding. Secondly, direct *N* ^9^ substitution, or tautomerization arising from *N* ^7^ substitution in some cases, leads to a dramatic reduction in affinity due to loss of a critical *N* ^9^-H···Val46 hydrogen bond, combined with the limited space available around the *N* ^9^ position. The water-filled pocket under the *N* ^7^ position is significantly more tolerant of substitution, with a hydroxyl ethyl 8-MG derivative attached to *N* ^7^ (compound **21a**) exhibiting an affinity for the *apo* enzyme comparable to the parent compound (*K_D_* ∼ 12 µM). In contrast to 8-MG, however, **21a** displays competitive binding with the ATP cofactor, as judged by NMR and SPR analysis. The 1.85 Å X-ray structure of the *Sa*HPPK/**21a** complex confirms that extension from the *N* ^7^ position towards the Mg^2+^-binding site, which affords the only tractable route out from the pterin-binding pocket. Promising strategies for the creation of more potent binders might therefore include the introduction of groups capable of interacting with the Mg^2+^ centres or Mg^2+^ -binding residues, as well as the development of bitopic inhibitors featuring 8-MG linked to a moiety targeting the ATP cofactor binding site.

## Introduction

Antibiotic resistance is rapidly emerging as one the most significant health challenges of this century [1]. In Europe alone, 25,000 deaths were reported in 2007 as a result of antimicrobial resistance, with an estimated cost of €1.3 billion per year [2]. Compounding this problem is the fact that antibiotic drug discovery is on the decline –a reflection of the considerable challenges associated with identifying both viable new targets as well as drugs to target them, but also a general lack of interest from large pharmaceutical companies. Most alarmingly, Methicillin-resistant *S. aureus* (MRSA) has evolved globally into a range of strains with varying phenotypes [3]. It has become resistant to both oxacillin and erythromycin, and resistance to levofloxacin is reported to be on the rise [4]. Community-acquired MRSA (caMRSA) is a relatively recent threat among patients without conventional risk factors. The epidemic USA300 strain of caMRSA is exceptionally virulent due to high levels of alpha toxin and the phenol-soluble modulins [4]; remarkably, it accounts for over half of all illnesses caused by the entire range of *S. aureus* species.

Logical targets for antimicrobials are essential enzymes that are unique to microorganisms, of which those of the folate biosynthesis pathway are prime examples. Folate is essential for the growth of all living cells, with the reduced form, tetrahydrofolate, used in the biosynthesis of thymidine, glycine and methionine. However, only bacteria and lower eukaryotes synthesize folate *de novo*; mammals and higher eukaryotes obtain it from their diet by active transport. The folate pathway enzymes, dihydropteroate synthase (DHPS) and dihydrofolatereductase (DHFR) are the targets for the sulfa drugs and Trimethoprim, respectively, which are used to treat diseases such as malaria, pneumocystis pneumonia (PCP), and, more recently, caMRSA infections.

It is well established that point mutations in pathogenic DHPS and DHFR genes contribute to widespread resistance to the aforementioned drugs. Recently, structure-based investigations have identified new inhibitors of DHPS that bind to the pterin site, remote from the sulpha drug site [5], as well as a new lead candidate for inhibiting the quadruple mutant DHFR enzyme conferring resistance in *Plasmoidium falciparum* [6]. These studies exemplify the application of modern drug discovery approaches to old targets as a means of generating potential new antibiotics.

**Figure 1:**
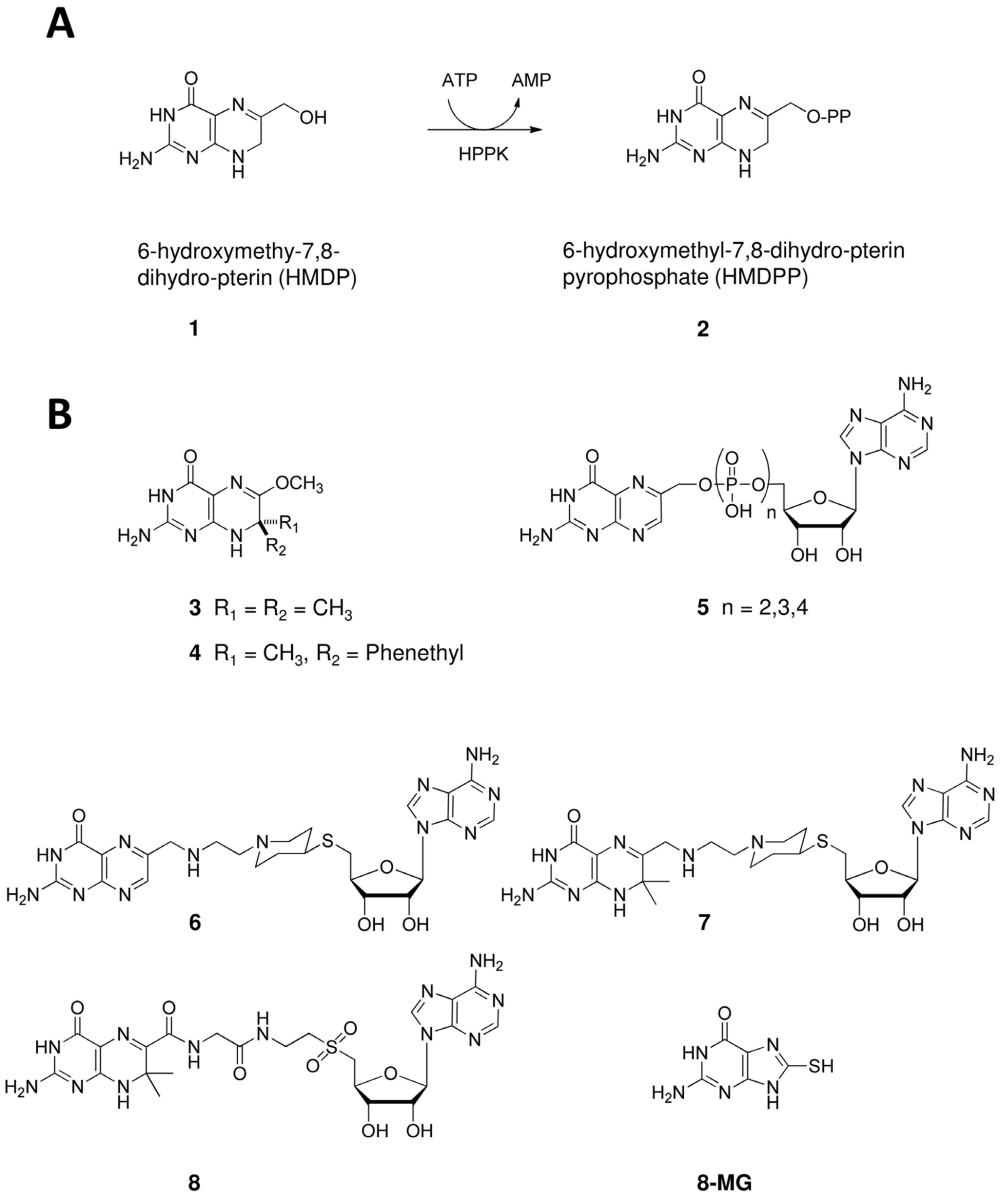
HPPK function and known inhibitors. A) HPPK catalysis. B) Known inhibitors of HPPK.

An alternative approach to combating resistant isolates is the development of inhibitors for as-yet-to-be-drugged enzymes within the folate pathway. Hydroxymethyl-pterin pyrophosphokinase (HPPK) is one such enzyme, responsible for catalysing the transfer of a pyrophosphate group from the ATP to the pterin substrate, 6-hydroxymethyl-7,8-dihydropterin (HMDP) (Fig. 1A). HPPK structures from many microbial sources have been solved (*E. coli, H. influenza, S. pneumoniae, S. cerevisiae, Y. pestis, F. tularensis* and *S. aureus*) [7]–[14]. All have a thioredoxin-like fold containing the binding sites for both the substrate and the ATP cofactor. X-ray structural studies have revealed that major conformational changes, particularly in loop L3, occur throughout the catalytic cycle [15]. Structural and kinetic studies [16]–[20] have also established that ATP binds (*K_D_* = 2.6–4.5 µM) prior to the substrate, which binds with sub-micromolar affinity. The pterin stacks between two highly conserved aromatic residues (Tyr or Phe) and both the substrate and cofactor are fixed in position by a multitude of hydrogen bonds; in total, they interact with 26 separate residues.

While much is known about the structure of HPPK, very few small molecule inhibitors have been developed (Fig. 1B). The gem-dimethyl- and 7-phenethyl-substituted pterin analogues, **3** and **4**, were reported to be HPPK inhibitors over three decades ago by Woods [21]. They have since been crystallized bound to the *E. coli* enzyme [11], [22], and **3** was utilized in a number of structural studies aimed at understanding the catalytic trajectory of HPPK [23], [24]. Recent inhibitor design has included the production of bitopic ligands featuring pterin coupled to adenosine via mono- through to tetra-phosphate linkers (**5**), with the longest linker providing the best affinity (*K_D_* = 0.47 µM) and inhibition (IC_50_ = 0.44 µM) [18]. Bitopic ligands featuring a more drug-like piperidine bridge (**6**) [20], or gem-dimethyl pterin in combination with a piperidine or amide-sulphone linker (**7** [20] and **8** [25]), have also been reported, however no gain in potency has been achieved (**8** did, however, display a novel binding mode in which the base was flipped).

**Figure 2:**
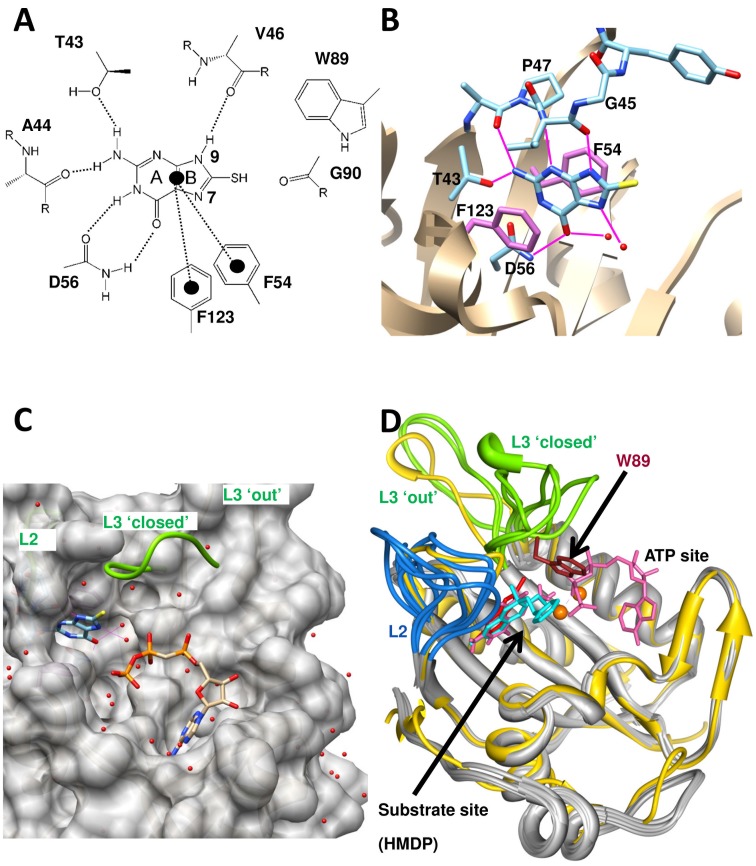
Structure of SaHPPK in complex with 8-MG. A–C) Structure of *Sa*HPPK (PDB:1QBC) in complex with 8-MG. A–B Intermolecular interactions between 8-MG and *Sa*HPPK. C) Surface representation of *Sa*HPPK showing the bound 8-MG (blue) overlayed with the closed loop L3 (green) and the bound AMPCPP as observed in the *Ec*HPPK/HMDP/AMPCPP (PDB:1Q0N) complex. D) Ribbon representation of the loop structure of several *Ec*HPPK structures overlayed with *Sa*HPPK (yellow) in complex with 8-MG (red) to illustrate the range of conformations in loops L2 and L3. The interaction of the Trp89 (brown) and the phenethyl inhibitor (cyan) is highlighted (PDB:1DY3) and the position of the HMDP (pink) and AMPCPP (pink) from *Ec*HPPK/HMDP/AMPCPP (PDB:1Q0N). Images were produced using the UCSF Chimera package (www.cgl.ucsf.edu/chimera).

Very recently, we showed that the simple guanine derivative, 8-mercaptoguanine (8-MG), is able to inhibit HPPK from *S. aureus* (*K_D_* ∼11 µM, IC_50_ = 41 µM) through interaction with the HMDP pocket [8]. Binding was found to be non-competitive with either the cofactor (ATP) or its non-hydrolyzable analogue, AMPCPP, as judged by both surface plasmon resonance (SPR) and isothermal titration calorimetry (ITC) analysis. A 1.65 Å resolution X-ray crystal structure revealed a high degree of stereo-electronic complementarity between 8-MG and the HMDP-binding pocket, together with an extensive network of hydrogen bonds, accounting for the unusually high binding affinity of the small 8-MG molecule (183 Da) (Fig. 2A, B). Most intriguingly, NMR analysis on the 8-MG/AMPCPP ternary complex provided compelling evidence that the SH group of 8-MG interacts with the L3 loop of *Sa*HPPK, locking it onto a “closed” conformation above the active site [26].

Herein, we report the results of a study interrogating the chemical space available within the active site of *Sa*HPPK and the chemical developability of 8-MG as a HPPK inhibitor. As part of this study, a series of *N* ^7^- and *N* ^9^-substituted 8-MG analogues have been synthesized, and their interaction with *Sa*HPPK examined using a combination of SPR, ITC, NMR spectroscopy and X-ray crystallography, in order to determine which of these positions are amenable to the lead optimization extension strategy. Additionally, a small number of *C* ^8^-sustituted analogues have been studied to allow further assessment of the relative importance of the SH group of 8-MG to the overall binding characteristics of this compound.

## Results and Discussion

### Structure-based Hypotheses and Design of 8-MG Analogues

As shown in Figure 2A and B, the pyrimidine heterocycle (ring A) of 8-MG is “perfectly tailored” for the pterin-binding pocket of HPPK, as evidenced by full saturation of all hydrogen donors and acceptors, and the sandwiching of the ring between the two phenylalanine residues, Phe54 and Phe123. This ring was therefore considered of limited value as a site for further chemical modification aimed at improving binding affinity and potency. Instead, our efforts focused on exploring the effects of substitution at the *N* ^7^, *C* ^8^ and *N* ^9^ positions of ring B.

Predicting the likely outcome of substituent changes/additions to 8-MG is complicated by the fact that the L2 and L3 catalytic loops in HPPK can adopt a diverse range of conformations, leading to drastic changes in the microenvironment of ring B (Fig. 2D) (loops L2 and L3 are also inherently dynamic in the *apo* and cofactor-bound states on the micro to millisecond timescale [23], [8]). The 8-MG/*Sa*HPPK X-ray structure (PDB: 3QBC) itself displays an extended L3 loop conformation [8], and is therefore limited in guiding modelling and structure-based design of 8-MG analogues from the *N* ^7^, *C* ^8^ and *N* ^9^ positions. In the first instance, we therefore chose to explore the effect of replacing the mercapto group of 8-MG with a variety of other substituents (compounds **10a**–**10f**, Table 1) in order to probe tolerance to substitution at this position. In part, this was performed in order to test our hypothesis (based on earlier ^15^N chemical shift and NMR relaxation measurements [8]), that Gly90 or Trp89 at the tip of the L3 loop form a favorable contact to the mercapto group at the *C* ^8^ position, which serves to fix L3 into a “closed” conformation resembling that observed in the ternary complex of *E. coli* HPPK, HMDP and AMPCPP (PDB: 1Q0N) (Fig. 2C) [15].

**Table 1:** Structures of *C* ^8^, *N* ^9^ and *N* ^7^-substituted guanine analogues and their binding affinities to *Sa*HPPK, as determined by SPR.

*C* ^8^-substituted analogues			
Compound	R_1_	*K*_D_ a (µM)	*K*_D_ b (µM)
10a	NHMe	No binding	108±5
10b	SMe	No binding	nd^c^
10c	Me	159±1	nd^c^
10d	Br	248±3	nd^c^
10e	OH	257±5	nd^c^
10f	N-morphilino	246±3	nd^c^
***N*** **^9^-substituted analogues**			
**Compound**	**R_2_**	***K*** **_D_** a **(µM)**	***K*** **_D_** b **(µM)**
15a	Me	194±5	410±10
15b	Et	190±10	340±10
15c	Bn	106±2	201±6
15d	CH_2_Bn	145±4	510±20
***N*** **^7^-substituted analogues**			
**Compound**	**R_3_**	***K*** **_D_** a **(µM)**	***K*** **_D_** b **(µM)**
21a	CH_2_CH_2_OH	12.3±1	130±10
21b	CH_2_COOH	No binding	140±4
21c	CH_2_CH_2_NH_2_	26.4±3	160±20
21d	CH_2_CH_2_CH_2_NH_2_	25.9±2	121±6
21e	CH_2_CH_2_NHC(NH)NH_2_	30±4	No binding

a in the presence of 10 mM Mg^2+.^

b in the presence of 10 mM Mg^2+^/1 mM ATP.

c nd: not determined.

Our substituent choices for the *N* ^9^ position were inspired by the structure of the ternary complex of *E. coli* HPPK with the phenethyl HMDP analogue (2-amino-6-methoxy-7-methyl-7-phenethyl-7,8-dihydropterin) and AMPCPP (PDB:1DY3) [22]. Within this structure, the phenyl ring of the substrate analogue makes two hydrophobic intermolecular interactions; one edge-on to Trp89 in loop L3 and the other to the side-chain of Leu45 (Val46 in *S. aureus*) in loop L2. From an overlay of 1DY3 and 3QBC (Fig. 2D), it was conjectured that the appendage of a hydrophobic group to the *N* ^9^ position of 8-MG could afford similarly favorable interactions with side-chains present in loops L2 and L3. Four hydrophobic substituents of increasing size (CH_3_, C_2_H_5_, CH_2_C_6_H_5_, CH_2_CH_2_C_6_H_5_) were thus chosen for investigation (compounds **15a–15d**, Table 1). In order to deliver a stronger binder, it was recognized that any favorable interaction(s) afforded by these groups would have to more than compensate for the loss of the hydrogen-bond between the *N* ^9^ H group and the Val 46 carbonyl in the *Sa*HPPK/8-MG complex (Fig. 2A, B).

Analysis of the *Sa*HPPK/8-MG crystal structure revealed a water-filled pocket proximal to the *N* ^7^ position (Fig. 2B, C). Given the hydrophilic nature of this region, it was postulated that attachment of a suitable polar substituent might enhance binding through provision of additional interactions with the polar side-chains and/or bound waters present, coupled with entropically-favorable water displacement. A small series of 8-MG analogues featuring alcohol, amine and guanidinium pendants attached to *N* ^7^ were therefore included within our test set (compounds **21a–21e**).

### Synthesis of 8-MG Analogues

8-(Methylamino)guanosine, **9**, synthesized as described in the literature [27], was hydrolyzed using 1 M HCl to afford the first of the test compounds, 8-(methyamino)guanine (**10a**). All other derivatives with *C* ^8^ substitution (**10b**–**10f**) were commercially sourced.

**Figure 3:**
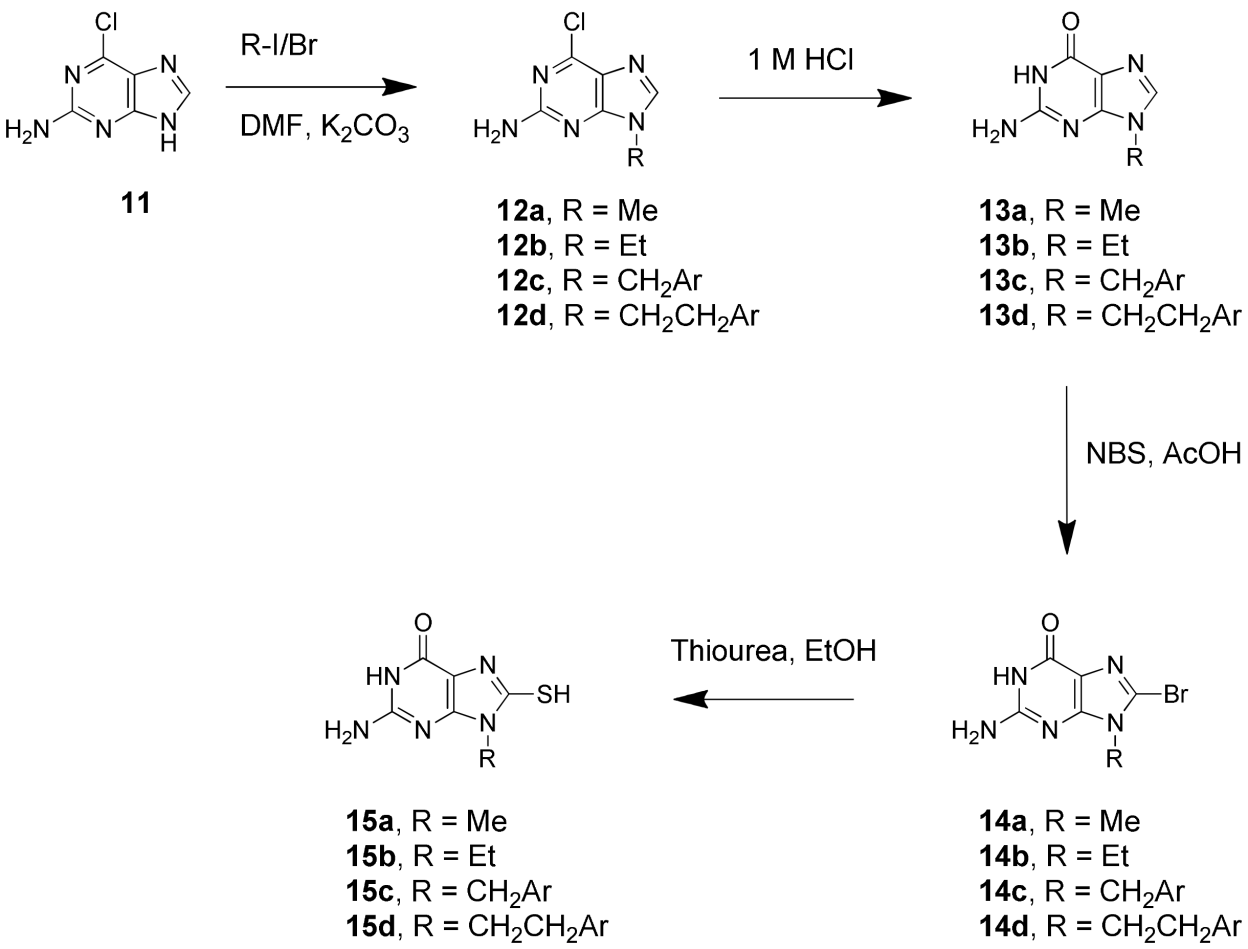
Synthetic scheme for N^9^-subtituted 8-MG analogues.

The synthetic routes to *N* ^9^-substituted guanines are well-established, in part because of the use of the *N* ^9^-substituted drugs, acyclovir and ganciclovir, in the treatment of herpes virus infections [28]. An expedient synthesis of the *N* ^9^ *-*methyl guanine from 2-amino-6-chloropurine, exploiting the *N* ^9^-directing effect of the chloro-substituent, has been previously reported and involved alkylation with methyl iodide followed by hydrolysis to install the oxo group [29]. We found this method could also be employed to provide ethyl, benzyl and 2-phenethyl substituents at the *N* ^9^-position (Fig. 3). Transformation of **13a** to the 8-mercapto derivative **15a** had been previously been demonstrated by bromination at *C* ^8^ to provide **14a** [30], followed by treatment with thiourea [31]. We found this similar transformation could be applied to our other derivatives providing the brominated analogues **14b–d** and the SH-containing target compounds, **15b–d**.

**Figure 4:**
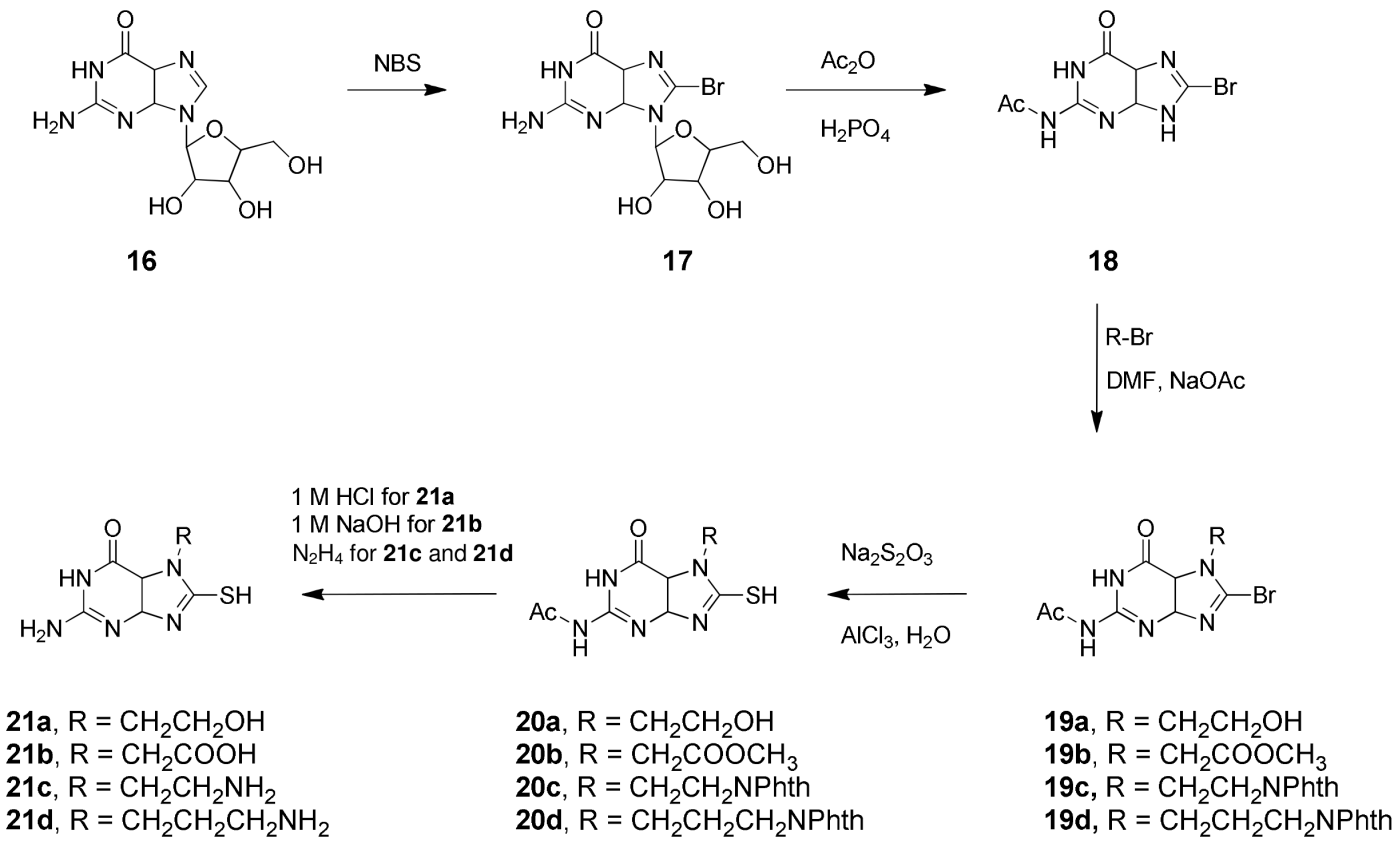
Synthetic scheme for N^7^-subtituted 8-MG analogues.

**Figure 5:**
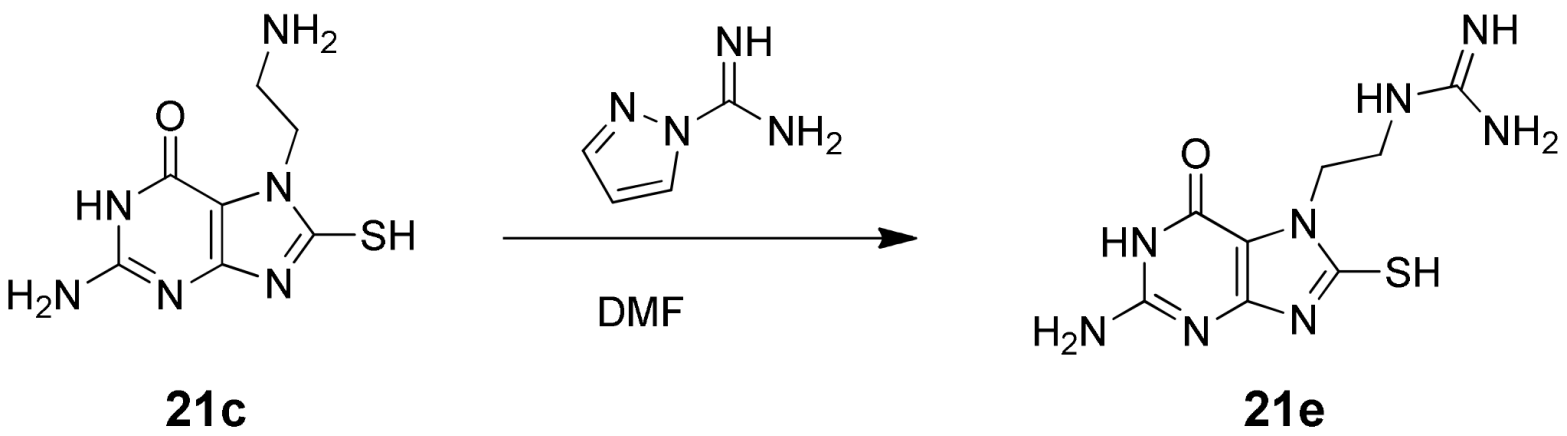
Synthetic scheme for compound 21e.

The *N* ^7^-substituted 8-MG analogues were prepared via alkylation of 8-bromo-N^2^-acetylguanine (**18**), formed in two steps from guanosine (**16**) according to a literature method [32], [33] (Fig. 4). Benzylation of **18** at *N* ^9^ with benzyl bromide has been reported previously under conditions that required no base [32]. We found that alkylation with other reagents proceeded well when the pH of the reaction mixture was adjusted to 3. These reactions generally yielded a *ca.* 1∶1 mixture of *N* ^7^- and *N* ^9^-substituted isomers, from which the desired *N* ^7^-alkylated intermediates were isolated following either silica gel or preparative-scale reverse-phase HPLC. Installation of the 8-mercapto group was then achieved by reaction with sodium thiosulfate in the presence of a catalytic quantity of aluminium-trichloride [34], and the target 8-MG analogues isolated following removal of any protecting groups under the appropriate conditions (Fig. 4). Compound **21e**, featuring an ethyl guanidinium group, was prepared from the amino analogue, **21c**, through reaction with pyrazole carboxamidine (Fig. 5).

All final compounds were purified by preparative HPLC to >95% purity.

### SPR and ITC Analysis of Binding

Initially, the binding of each of the test compounds to *Sa*HPPK was quantitatively assessed using SPR (Fig. S1, S2). Compared with the parent compound (8-MG), SPR data for the synthesized analogues did not appear to be compromised by their solubility in aqueous buffer at the maximum concentration used (126 µM). Moreover, all sensorgrams (Fig. S1 and S2) were of high quality and consistent with near perfect 1∶1 stoichiometric binding of analogues. Table 1 lists the estimated equilibrium dissociation constants (*K_D_*)_._ It is clear from the data that replacement of the 8-mercapto group is highly detrimental to binding; compounds **10a** and **10b**, featuring -NHCH_3_ and -SCH_3_ groups at the *C* ^8^ position, did not bind *Sa*HPPK at all (although binding of compound **10a** was detected (*K*_D = _108 µM) in the presence of saturating amounts of ATP), whilst all other *C* ^8^-substiuted analogues exhibited 15–20-fold lower *K*_D_ values than 8-MG. This supports the hypothesis that the 8-mercapto group of 8-MG aids binding through the formation of one or more favorable interactions with *Sa*HPPK. The precise nature of this/these interaction(s) remain unclear, however the considerably inferior binding affinity of the *C* ^8^-OH analogue (**10e**) suggest that it is unlikely to be a simple hydrogen bond to a loop L3 residue, as we speculated might be the case earlier [8].

The 8-MG derivatives with simple hydrophobic substituents at the *N* ^9^ position (**15a**–**d**) exhibited 10-20-fold lower affinities for *Sa*HPPK, indicating that any potential positive contribution to binding afforded by these groups is not sufficient to make up for the loss of the intramolecular *N* ^9^-H·Val46 carbonyl hydrogen bond. Extension of the 8-MG scaffold via the *N* ^9^ position, therefore, does not appear to be a promising strategy for lead optimization.

Of the *N* ^7^-substituted 8-MG analogues, compound **21a**, with an ethyl alcohol substituent, displayed comparable affinity to 8-MG (*K*_D_ ∼12 µM), whilst the analogues with amine and guanidinum pendants (**21c**–**21e**) displayed slightly weaker binding to *Sa*HPPK; the carboxylate pendant-bearing analogue, **21b**, did not bind. This indicates that addition of substituents at the *N* ^7^ position are tolerated, and that extension from this ring position is likely the most promising avenue for future development of more potent 8-MG analogues. It should be noted, however, that in contrast to 8-MG, the binding of compounds **21a, 21c** and **21d** was found to be 10-15-fold weaker under saturating ATP conditions, suggesting extensions from ring B into the space towards the Mg^2+^ centres and the ATP binding site leads to competitive binding with the ATP cofactor. Any future lead optimization studies will need to bear this in mind.

To corroborate the ligand binding affinities determined by SPR, and to determine the enthalpic and entropic contributions to the free energy of binding, ITC experiments were performed for selected compounds (**21a** and **21c**–**21e**) (Table 2, Fig. S3). *K*_D_ values in the 17–35 µM range were obtained, in excellent agreement with the values obtained by SPR. Interestingly, compound **21a**, which yielded a similar *K*_D_ value to 8-MG (16.7 µM *vs.* 12.8 µM), showed a lower binding enthalpy than 8-MG, but its binding to *Sa*HPPK was associated with a much lower entropic penalty. Given that **21a** has more rotatable bonds than 8-MG, this may suggest that binding of the latter may lead to a greater degree of immobilization of the catalytic loops within *Sa*HPPK. To investigate the factors contributing to the free energy of binding, we solved the structure of *Sa*HPPK in complex with **21a**.

**Table 2:** Thermodynamic parameters for the binding of selected compounds to *Sa*HPPK as determined by ITC^a^.

Compound	ΔH	TΔS	ΔG	N	*K*_D_ (µM)
	(kCal mol^−1^)	(kCal mol^−1^)	(kCal mol^−1^)		ITC
8-MG^b^	−19.6±3.4	−12.9±3.5	−6.7±0.2	1.0±0.06	12.8±3.4
21a	−10.5±0.6	−4.1±0.5	−6.4±0.1	0.8±0.10	16.7±3.5
21c	−5.8±0.1	0.4±0.2	−6.2±0.1	1.2±0.02	22.4±2
21d	−6.2±0.4	−0.2±0.5	−6.0±0.1	1.3±0.05	34.3±6.2
21e	−4.3±0.1	1.7±0.2	−6.0±0.1	1.4±0.02	35.1±4.8

a Values are the means ± the standard deviation for at least three experiments. All ITC and SPR experiments were performed at 298 K and 293 K, respectively. ^b^ data from Chhabra *et al* PlosONE 2012.

### X-ray Structure of SaHPPK in Complex with Compound 21a

**Figure 6:**
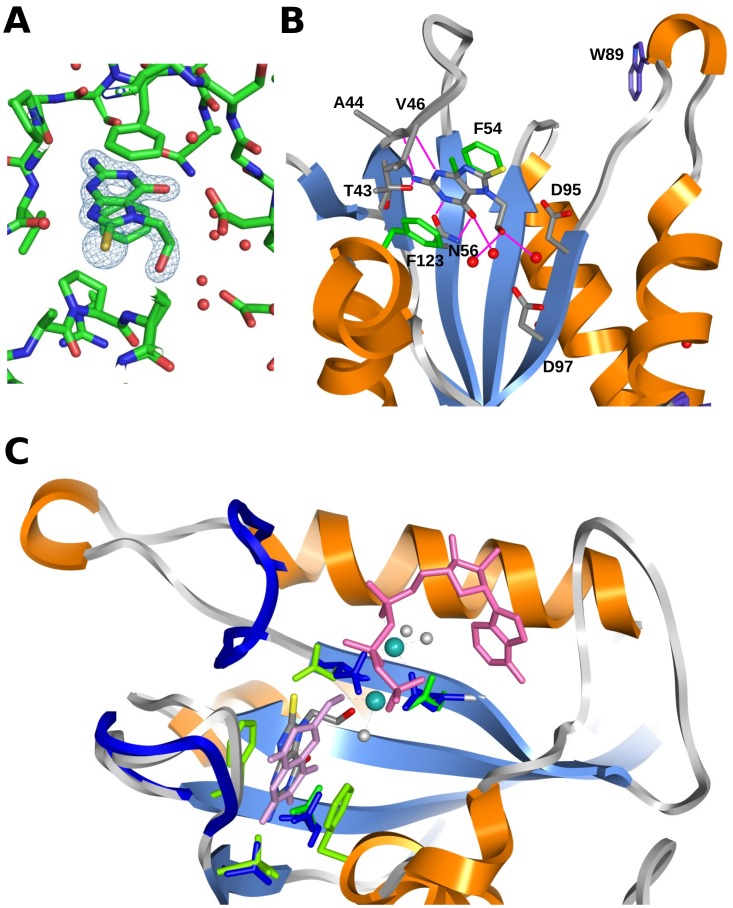
Structure of SaHPPK in complex with 21a. (A) 2Fo-Fc electron density map of the pterin binding site, contoured at 2.0 sigma showing density for **21a**. B) Detail of intermolecular interactions from **21a** to *Sa*HPPK and two bound waters. C) Superposition of the *Ec*HPPK/HMDP/AMPCPP (PDB:1QON) structure. Selected loops and sidechains of *Ec*HPPK are shown (blue) along with the bound HMDP (plum) and AMPCPP (pink), two bound magnesium ions (dark cyan) and the oxygen atoms of coordinating waters (grey). *Sa*HPPK/**21a** is shown colored as in B) with selected sidechains shown (green). Images were produced using the UCSF Chimera package (www.cgl.ucsf.edu/chimera) and PyMOL (Delano Scientific).

Attempts were made to co-crystallize each of the strongest binding compounds (**21a** and **21c**–**21e**) with *Sa*HPPK, however diffraction quality crystals could only be obtained for compound **21a**. These provided excellent quality electron density data, and a high-resolution X-ray structure (1.85 Å) of the *Sa*HPPK/**21a** binary complex (Fig. 6A) determined via molecular replacement (crystal data and details of the data collection and refinement are provided in Table 3). A head-to-tail protein dimer was found in the asymmetric unit, similar to that observed for the earlier *Sa*HPPK/8-MG structure (PDB: 3QBC) [8], with the ligand bound to the pterin sites of both protein monomers. The ethyl alcohol pendant projects into the space leading towards the Mg^2+^ binding site, making two hydrogen bond contacts with a pair of bound water molecules (Fig. 6B). Presumably, these interactions in part compensate for the loss of the hydrogen bond between the *N* ^9^-H of 8-MG and the backbone carbonyl of Val46, which occurs as a consequence of the tautomerization accompanying alkylation at the *N* ^7^ position. A water molecule found in the cavity under *N* ^7^ in the *Sa*HPPK/8-MG structure has been displaced in the *Sa*HPPK/**21a** structure, and there is a tightly bound water between the hydroxyethyl and Asp97 which orients Asp97 in a similar position to that found of Asp97 of the *Ec*HPPK/AMPCPP/HMDP structure (where Mg^2+^ sits). Superposition of the *Sa*HPPK/**21a** structure with that of *Ec*HPPK/AMPCPP/HMDP (PDB: 1QON) [15] indicates that if Mg^2+^ ions and ATP were simultaneously bound, the oxygen of the hydroxyethyl pendant of **21a** is displaced by ∼1 Å and would lie only 1.5–1.6 Å from one of the metal ions, which is considerably less than the Mg-O bond length observed in the 1QON structure (2.1 Å) and sterically unfavorable (Fig. 6C). This is the likely reason for the cofactor and metal competition observed for **21a**.

**Table 3:** X-ray structure data processing and refinement statistics.

Spacegroup	Monoclinic, *P*21
X-ray source	MX2, Australian Synchrotron
Detector	ADSC Quantum 315
Wavelength (Å)	0.9537
Unit-cell parameters (Å, °)	a = 36.6, b = 75.7, c = 51.4, α = γ = 90.0, β = 99.7
Diffraction data	
Resolution range (Å)	42.10–1.85 (1.90–1.85)
No. of unique reflections	23175 (1543)
Matthews coefficient, *V*_M_ (Å^3^ Da^−1^)	1.95
Solvent content (%)	36.9
Completeness (%)	98.3 (97.6)
Data redundancy	6.9 (6.6)
Mean I/σ(I)	10.5 (3.0)
R_merge_ (%)*	14.7 (57.5)
R_p.i.m._ (%)#	5.9 (23.2)
Refinement (50.6–1.85?Å)	
*R*_free_ (%)	26.4
*R*_cryst_ (%)	20.9
Size of *R*_free_ set (%)	5
Protein residues (dimer)	320
Inhibitor Molecules	2
Water molecules	163
*RMSD from ideal values:*	
Bond lengths (Å)	0.016
Bond angles (°)	1.819
Ramachandran plot	
Residues in most favoured regions (%)	97.3
Residues in allowed regions (%)	2.4
Residues in disallowed regions (%)	0.3
Estimated coordinate error (Å)	0.179
Mean B factors (Å^2^)	15.1

* R_merge_ = ΣhΣi |*I*i(h) - <*I*(h)>|/ΣhΣi*I*i (h),

# R_pim_ = Σh [1/(N-1)]1/2 Σi |*I*i(h) - <*I*(h) >|/ΣhΣi*I*i (h).

Values in parentheses refer to the outer resolution shell (1.74–1.65 Å).

Where *I* is the observed intensity, <*I*> is the average intensity of multiple observations from symmetry-related reflections, and N is redundancy.

R_value_ = _jjFoj _ jFcjj/_jFoj, where Fo and Fc are the observed and calculated structure factors. For R_free_ the sum is done on the test set reflections (5% of total reflections), for R_work_ on the remaining reflections.

### Heteronuclear NMR Analysis of Compound 21a Binding to SaHPPK

**Figure 7:**
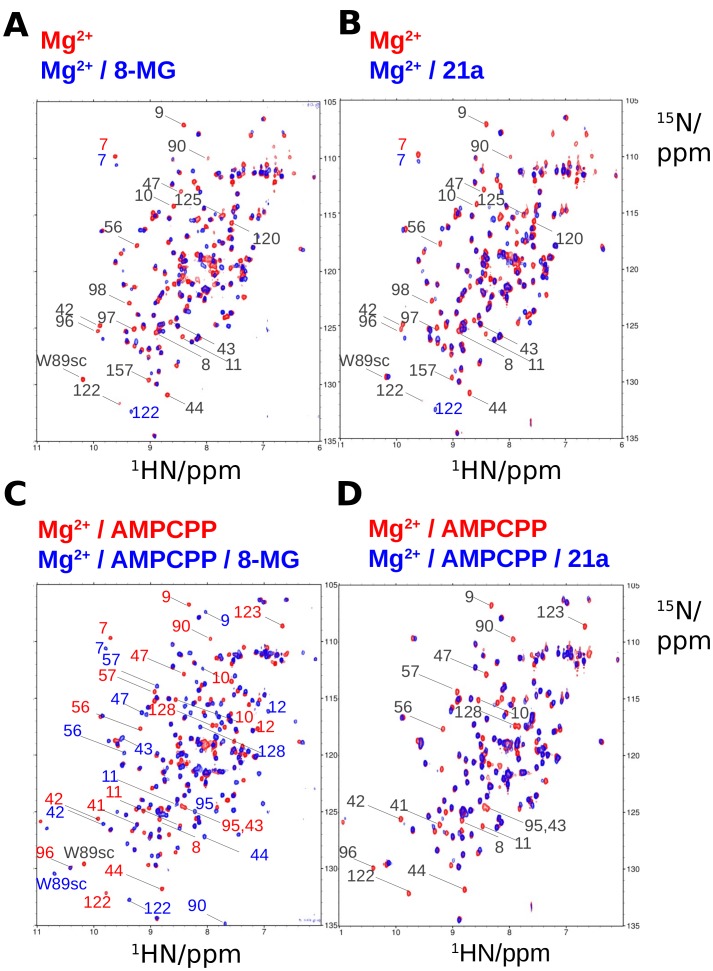
Comparing the binding of 21a and 8-MG to apo and cofactor bound SaHPPK as judged by 2D NMR. A–B) Binding of 8-MG and **21a** to magnesium bound *Sa*HPPK are very similar. C–D) Binding of 8-MG and **21a** to the AMPCPP bound *Sa*HPPK are very different. Figures A and C are adapted from data in Figure 6A in [8]. The concentration of ^15^N-labelled *Sa*HPPK was ∼100 µM in all cases. The concentration of magnesium, AMPCPP, 8-MG and **21a** was 10 mM, 1 mM, 0.6 mM and 0.6 mM respectively. The assignment of selected substrate site peaks are shown to highlight the effects of binding of the two compounds on the NMR spectra. The sidechain Hε1–Nε1 peak of Trp89 is labelled as W89sc.

Titration of **21a** into ^15^N-labelled *apo* (data not shown) and magnesium-loaded *Sa*HPPK enzyme led to broadening and disappearance of several, common peaks in the 2D ^15^N HSQC NMR spectrum (Fig. 7A, B), which is characteristic of the intermediate exchange timescale, and indicates that binding of **21a** is not magnesium-dependent. This is similar to what was observed for the binding of 8-MG to *Sa*HPPK (Fig. 7A) and is consistent with the fact that density characteristic of magnesium was not observed in the X-ray crystal data of the *Sa*HPPK**/21a** complex.

The observed intermediate exchange regime for the binding of 8-MG and **21a** is possibly dictated in part by the slow µs-ms timescale motion of loop L3 [23], [24]. While the spectra (Fig. 7A, B) appear to be very similar, however, closer inspection reveals that the sidechain Hε1–Nε1 peak of Trp89 (in loop L3) is only perturbed in the 8-MG bound spectrum (Fig. 7A). This is mechanistically interesting and may indicate that this region of loop L3, adjacent to the substrate-binding loop L2, is involved in binding of 8-MG but not **21a**. Following on from this, the observed larger entropic penalty to the free energy of binding of 8-MG as compared to **21a** (Table 2) may derive in part from this increase in loop L3 rigidity in the presence of 8-MG, whilst the more favorable enthalpic contribution likely reflects the formation of the *N* ^9^-H Val46 intermolecular hydrogen bond (as observed in the X-ray structure). Reduced loop L3 involvement in **21a** binding, on the other hand, is likely a result of the loss of the *N* ^9^-H Val46 intermolecular hydrogen bond (due to tautomerization from substitution at *N* ^7^), which would reduce any dampening of the adjacent loop L2 dynamics. Ligand-induced loop L2 and L3 dampening can be detected and investigated directly by NMR, but in order to do this the NMR timescale needs to be shifted out from the intermediate regime. This was previously accomplished by binding 8-MG to the AMPCPP bound *Sa*HPPK enzyme [8]. The results of our heteronuclear NMR spin relaxation studies reported therein revealed dampening of loop L2 and L3 motion on the fast timescale compared to the apo or the AMPCPP (or ATP)-bound *Sa*HPPK enzyme. Unfortunately, it was not possible to investigate the enzyme dynamics in the same way for the binding of **21a** as it was found to be competitive with AMPCPP. From a comparison of the X-ray structures of the **21a**/*Sa*HPPK binary complex (this work) with our previous 8-MG/*Sa*HPPK binary structure [8], the expulsion of a bound water underneath *N* ^7^ is likely to be thermodynamically favorable for the binding of **21a**, and in line with the observed reduced entropic penalty. This may be the reason for the reduced entropic penalty associated with binding of the **21a–c** series as a whole; see Table 2.

Repeating the titration in the presence of a saturating amount of the cofactor analogue, AMPCPP (*K*_D_ = 3 µM), led to broadening of the same pterin site signals and the peak corresponding to the sidechain of Trp89 displayed very little change, in accord with the lack of involvement of this residue in **21a** binding, as described previously. In accordance with the SPR data, however, cross peaks in slow exchange, characteristic of formation of a ternary complex (observed in our earlier study of the interaction of 8-MG with *Sa*HPPK in the presence of AMPCPP (Fig. 7C)) were absent (Fig. 7D), indicating that binding of **21a** to *Sa*HPPK is competitive with AMPCPP.

### Conclusion

8-MG represents a promising scaffold for the potential development of a new antibiotic drug targeting the folate pathway enzyme, HPPK. This study has shown that the 8-mercapto group plays a pivotal role in binding, and ought to be maintained in any future lead optimization studies. Extension from the *N* ^9^ position within ring B leads to a dramatic loss of affinity and is therefore not a viable site for chemical modification. Substitution at the *N* ^7^ position, however, is tolerable, as exemplified by *N* ^7^-hydroxyethyl-8-MG (**21a**), which was found to bind *Sa*HPPK with comparable affinity to the parent compound. An important caveat is that extension into the space surrounding the *N* ^7^ atom leads to competitive binding with the ATP cofactor. To provide a meaningful enhancement in potency, future studies will therefore need to focus on the development of *N* ^7^ pendants that interact strongly with the residues surrounding this pocket. This could include the introduction of groups to bind to the absolutely conserved metal-binding residues, Asp95 and Asp97, within the *apo* form of the enzyme. An alternate route to an increase in potency could involve changing the nature of ring B of the 8-MG core such that the *N* ^9^-H Val46 H bond is maintained whilst still allowing extension from the *N* ^7^ position towards the highly conserved metal-binding residues. We are currently investigating this approach.

Compared to the reported bitopic inhibitors for HPPK [18], [20], [25], both 8-MG and **21a** are less potent, yet they have better ligand efficiencies (*K*_D_ ∼10 µM over 12 and 15 heavy atoms, respectively, compared to *K*_D_ ∼ 3 µM over 40+ heavy atoms). 8-MG could potentially be linked to adenosine to provide a bitopic ligand with considerably enhanced affinity, though problems associated with linking two subsite binders as a route to higher affinity have been well documented [35], [36]. Ultimately, incremental step-wise chemical evolution of the 8-MG scaffold in a more conventional manner may prove the most efficient route to developing an inhibitor with superior pharmacodynamic and pharmacokinetic properties.

Finally, it is worth noting that it has recently been shown that 8-MG can also bind to the pterin pocket in DHPS, the adjacent, downstream enzyme to HPPK [37]. The chemical strategies described herein may therefore prove beneficial for the design of more potent DHPS inhibitors based on the 8-MG scaffold, and perhaps even for the development of agents capable of inhibiting multiple enzymes within the folate biosynthesis pathway.

## Methods

### Chemistry - General Methods

Melting points were determined on a Mettler Toledo MP50 melting point system and are uncorrected. The abbreviation *dec.* indicates that the compound decomposed at the specified temperature. ^1^H and ^13^C NMR spectra were recorded on a Bruker Ultrashield 400 Plus at 400 MHz and 101 MHz, respectively. Analytical HPLC was performed on a Waters Alliance 2690 fitted with a Waters 5996 PDA detector and a Phenomenex Luna C_8_ column (5 µm, 100 Å, 150 × 4.60 mm). Analyses were conducted using a gradient of 0 to 64% acetonitrile in water over 10 min with 0.1% trifluoroacetic acid (TFA) throughout. Preparatory HPLC was performed on a Waters Prep LC 4000 system fitted with a Waters 486 Tunable Absorbance Detector and either a Phenomenex Luna C_18_ (10 µm, 100 Å, 250 × 30 mm) column or a Phenomenex Luna C_8_ (10 µm, 100 Å, 50 × 21.2 mm) column. Low resolution mass spectrometry was performed on an Agilent 6120 single quadrapole LCMS system using electrospray ionization. High resolution mass spectrometry was performed on a Waters Premier XE time-of-flight mass spectrometer using electrospray ionization.

### Chemistry - Synthesis

#### 8-(Methylamino)guanine (10a)

A solution of 8-(methylamino)guanosine **9** (50 mg, 0.20 mmol) in 1 M HCl (10 mL) was refluxed for 2 h, then cooled to rt (room temperature). The precipitate was collected by filtration and resuspended in water (5 mL). This mixture was made basic by drop wise addition of 1 M NaOH whereupon the precipitate dissolved. Reverse phase chromatography (C18, 1% TFA in water) provided the title compound as a white solid (30 mg, quantitative). **Mp** 252–257°C (dec.), **^1^H NMR** (400 MHz, D_2_O) δ 2.66 (s, 3H).**^13^C NMR** (101 MHz, D_2_O) δ 164.2, 163.6, 162.5, 157.5, 116.2, 30.0. **LRMS** (ESI): *m/z*: 181.1 ([M+H]^+^100%). **HRMS** (ESI): observed *m/z:* 181.0837; calculated *m/z:* 181.0832 [M+H]^+^.

#### 9-Ethylguanine (13b)

A solution of 2-amino-6-chloropurine (1.00 g, 5.90 mmol) in DMF (10 mL) was treated with ethyl iodide (472 µL, 5.89 mmol) and K_2_CO_3_ (815 mg, 5.89 mmol). After stirring for 15 h at rt the solution was evaporated to dryness under reduced pressure and 2-amino-*N* ^9^-ethyl-6-chloropurine isolated by silica gel chromatography (CHCl_3_/MeOH, 95∶5). This material was refluxed in 1 M HCl (20 mL) for 2 h then cooled to rt. The resulting precipitate was collected by filtration, affording the title compound as a white powder (527 mg, 50%). **^1^H NMR** (400 MHz, DMSO-d_6_) δ 12.03 (s, 1H), 9.34 (s, 1H), 7.58 (s, 2H), 4.15 (q, *J* = 7.3 Hz, 2H), 1.43 (t, *J* = 7.3 Hz, 3H). **^13^C NMR** (101 MHz, DMSO-d_6_) δ 155.8, 153.1, 149.4, 136.7, 107.2, 34.0, 14.2. **LRMS** (ESI): *m/z*: 180.1 [M+H]^+^ (100%).

#### 9-Benzylguanine (13c)

A solution of 2-amino-6-chloropurine (1.00 g, 5.90 mmol) in DMF (10 mL) was treated with benzyl bromide (700 µL, 5.89 mmol) and K_2_CO_3_ (815 mg, 5.89 mmol), and stirred for 15 h at rt. The intermediate 2-amino-*N* ^9^-benzyl-6-chloropurine was isolated and hydrolyzed as described for the preparation of **13b,** to provide the title compound as a white powder (1.30 g, 90%). **^1^H NMR** (400 MHz, DMSO-d_6_) δ 11.79 (s, 1H), 9.18 (s, 1H), 7.41–7.29 (m, 7H), 5.35 (s, 2H). **^13^C NMR** (101 MHz, DMSO-d_6_) δ 155.8, 153.8, 150.0, 137.3, 135.3, 129.0, 128.4, 127.8, 108.8, 47.5. **LRMS** (ESI): *m/z*: 242.2 [M+H]^+^ (100%).

#### 9-Phenethylguanine (13d)

A solution of 2-amino-6-chloropurine (1.00 g, 5.90 mmol) in DMF (10 mL) was treated with 2-phenethyl bromide (798 µL, 5.89 mmol) and K_2_CO_3_ (815 mg, 5.89 mmol), and stirred for 15 h at rt. The intermediate 2-amino-*N* ^9^-(2-phenethyl)-6-chloropurine was isolated and hydrolyzed as described for the preparation of **13b** to provide the title compound as a white powder (1.35 g, 90%). **^1^H NMR** (400 MHz, DMSO-d_6_) δ 11.78 (s, 1H), 8.89 (s, 1H), 7.34–7.15 (m, 7H), 4.34 (t, *J* = 7.3 Hz, 2H), 3.15 (t, *J* = 7.3 Hz, 2H).**^13^C NMR** (101 MHz, DMSO-d_6_) δ 155.5, 153.6, 149.7, 137.1, 136.9, 128.6, 128.6, 126.8, 108.4, 45.5, 34.1.**LRMS** (ESI): *m/z*: 256.2 [M+H]^+^ (100%).

#### 8-Bromo-9-ethylguanine (14b)

A solution of compound **13b** (212 mg, 0.820 mmol) in glacial acetic acid (15 mL) was treated with *N*-bromosuccinimide (211 mg, 1.19 mmol). After stirring for 15 h at rt, the solution was poured into a mixture of ice (50 g) and water (100 mL). The precipitate was filtered, washed with water and methanol, then dried to provide the title compound as a yellow powder (122 mg, 58%). **^1^H NMR** (400 MHz, DMSO-d_6_) δ 10.66 (s, 1H), 6.58 (s, 2H), 3.96 (q, *J* = 7.2 Hz, 2H), 1.25 (t, *J* = 7.2 Hz, 3H). **^13^C NMR** (101 MHz, DMSO-d_6_) δ 155.5, 153.8, 152.0, 120.2, 116.8, 31.4, 14.5. **LRMS** (ESI): *m/z*: 258.1 [M+H]^+^ (100%).

#### 8-Bromo-9-benzylguanine (14c)

A solution of compound **13c** (455 mg, 1.89 mmol) in glacial acetic acid (30 mL) was treated with *N*-bromosuccinimide (453 mg, 2.55 mmol). After stirring for 15 h at rt, the product was isolated using the procedure described for **14b**, providing the title compound as a yellow powder (384 mg, 65%). **^1^H NMR** (400 MHz, DMSO-d_6_) δ 10.72 (s, 1H), 7.38–7.15 (m, 5H), 6.60 (s, 2H), 5.16 (s, 2H). **^13^C NMR** (101 MHz, DMSO-d_6_) δ 155.6, 154.0, 152.2, 135.8, 128.6, 127.7, 126.6, 120.8, 115.0, 45.4. **LRMS** (ESI): *m/z*: 320.1 [M+H]^+^ (100%).

#### 8-Bromo-9-phenethylguanine (14d)

A solution of compound **13d** (1.21 g, 4.57 mmol) in glacial acetic acid (90 mL) was treated with *N*-bromosuccinimide (1.10 g, 6.20 mmol). After stirring for 15 h at rt, the product was isolated using the procedure described for **14b**, providing the title compound as a yellow powder (914 mg, 60%). **^1^H NMR** (400 MHz, DMSO-d_6_) δ 10.68 (s, 1H), 7.49–6.89 (m, 5H), 6.59 (s, 2H), 4.15 (t, *J* = 7.8 Hz, 2H), 3.00 (t, *J* = 7.8 Hz, 2H). **^13^C NMR** (101 MHz, DMSO-d_6_) δ 155.5, 153.8, 151.9, 137.3, 128.6, 128.4, 126.6, 120.7, 115.0, 43.9, 34.3. **LRMS** (ESI): *m/z*: 334.2 [M+H]^+^ (100%).

#### 8-Mercapto-9-ethylguanine (15b)

A solution of compound **14b** (102 mg, 0.40 mmol) and thiourea (60 mg, 0.80 mmol) in EtOH (5 mL) was refluxed for 15 h. The solvent was removed *in vacuo* and the residue purified using reverse phase chromatography (C18, isocratic: 0.1% TFA in water) to afford the title compound as an off-white solid (51 mg, 60%). **Mp** 240–244°C (*dec*.), **^1^H NMR** (400 MHz, DMSO-d_6_) δ 12.71 (s, 1H), 10.87 (s, 1H), 6.66 (s, 2H), 4.01 (q, *J* = 7.1 Hz, 2H), 1.23 (t, *J* = 7.1 Hz, 3H). **^13^C NMR** (101 MHz, DMSO-d_6_) δ 155.8, 153.1, 149.4, 136.7, 107.2, 34.0, 14.2. **LRMS** (ESI): *m/z*: 212.1 [M+H]^+^ (100%), **HRMS** (ESI): observed *m/z:* 212.0603 [M+H]^+^; calculated *m/z:* 212.0601 [M+H]^+^, **RP-HPLC**: t_R_ 5.26 min, >98%.

#### 8-Mercapto-9-benzylguanine (15c)

A solution of compound **14c** (110 mg, 0.34 mmol) and thiourea (131 mg, 1.70 mmol) in EtOH (5 mL) was refluxed for 15 h. The solvent was removed *in vacuo* and the residue purified using reverse phase chromatography (C18, isocratic: 0.1% TFA in water) to afford the title compound as an off-white solid (65 mg, 70%). **Mp**>300°C (*dec*.), **^1^H NMR** (400 MHz, DMSO-d_6_) δ 12.87 (s, 1H), 10.94 (s, 1H), 7.41–7.15 (m, 5H), 6.64 (s, 2H), 5.21 (s, 2H). **^13^C NMR** (101 MHz, DMSO-d_6_) δ 164.7, 154.1, 150.8, 150.0, 136.4, 128.3, 127.3, 127.2, 103.7, 44.9. **LRMS** (ESI): *m/z*: 274.1 [M+H]^+^ (100%), **HRMS** (ESI): observed *m/z:* 274.0770 [M+H]^+^; calculated *m/z:* 274.757 [M+H]^+^, **RP-HPLC**: t_R = _7.31 min, >95%.

#### 8-Mercapto-9-phenethylguanine (15d)

A solution of compound **14d** (500 mg, 1.50 mmol) and thiourea (228 mg, 3.00 mmol) in EtOH (5 mL) was refluxed for 15 h. The solvent was removed *in vacuo* and the residue purified using reverse phase chromatography (C18, isocratic: 0.1% TFA in water) to afford the title compound as an off-white solid (260 mg, 60%). **Mp** 291–295°C (*dec*.), **^1^H NMR** (400 MHz, DMSO-d_6_) δ 12.76 (s, 1H), 10.90 (s, 1H), 7.35–7.21 (m, 5H), 6.65 (s, 2H), 4.18 (t, *J* = 7.8 Hz, 2H), 2.99 (t, *J* = 7.8 Hz, 2H). **^13^C NMR** (101 MHz, DMSO-d_6_) δ 164.1, 154.0, 150.8, 149.9, 138.0, 128.5, 128.4, 126.4, 103.6, 43.0, 33.1. **LRMS** (ESI): *m/z*: 288.2 ([M+H]^+^100%), **HRMS** (ESI): observed *m/z:* 288.09 [M+H]^+^; calculated *m/z:* 288.0914 [M+H]^+^, **RP-HPLC**: t_R = _7.9 min, >98%.

#### *N* ^2^-Acetyl-8-bromo-7-(2-hydroxyethyl)guanine (19a)

To a suspension of *N* ^2^-acetyl-8-bromoguanine (**18**) (100 mg, 0.37 mmol) in DMF (1 mL) was added 2-bromoethanol (50 µL, 0.70 mmol) and DIPEA (32 µL, 0.20 mmol). The reaction was heated at 100°C for 24 h with periodic addition of DIPEA in order to maintain the pH between 3 and 4. The solution was diluted with water (5 mL) and purified and subjected to reverse phase chromatography (C18, 0–4% ACN with 0.1% TFA in water) to isolate the title compound as a white solid (30 mg, 26%). **^1^H NMR** (400 MHz, DMSO-d_6_) δ 4.31 (t, *J* = 5.6 Hz, 2H), 3.72 (t, *J* = 5.6 Hz, 2H), 2.16 (s, 3H).**^13^C NMR** (101 MHz, CDCl_3_) δ 21a3.5, 156.5, 151.5, 147.4, 131.3, 113.7, 59.8, 49.4, 23.7. **LRMS** (ESI): *m/z*: 315 [M+H]^+^, (100%), 321 [M+H]^+^ (100%).

#### Methyl-8-bromo-(*N* ^2^-acetylguanin-7-yl)acetate (19b)

To a suspension of *N* ^2^-acetyl-8-bromoguanine (**18**) (1.00 g, 3.70 mmol) in dry DMF (5 mL) under N_2_ was added DIPEA (1.30 mL, 7.40 mmol) and methyl bromoacetate (386 µL, 4.10 mmol). The solution was stirred for 20 h at rt, the solvent removed *in vacuo*, and the residue was coevaporated with methanol (3×). The residue was chromatographed on silica gel (MeOH/DCM, 1∶19) to provide the title compound as a white solid (255 mg, 20%). **^1^H NMR** (400 MHz, DMSO-d_6_) δ 7.95 (s, 1H), 5.02 (s, 2H), 3.70 (s, 3H), 2.16 (s, 3H). **^13^C NMR** (101 MHz, DMSO-d_6_) δ 213.5, 168.1, 154.7, 148.9, 147.9, 140.1, 119.6, 52.5, 44.1, 23.7. **LRMS** (ESI): *m/z*: 345.9 [M+H]^+^ (100%), 347 [M+H]^+^ (100%).

#### *N*-2-(8-bromo-*N* ^2^-acetylguanin-7-yl)ethylphthalimide (**19c**)

A solution of *N* ^2^-acetyl-8-bromoguanine (**18**) (500 mg, 1.85 mmol), DIPEA (960 µL, 5.60 mmol) and *N*-(2-bromoethyl)phthalimide (560 mg, 1.85 mmol) in DMF (5 mL) were heated at 100°C under N_2_ for 15 h. The solvent was removed *in vacuo*, and the residue was coevaporated with methanol (3×). The crude mixture was purified using silica gel chromatography (petroleum spirits/ethylacetate/methanol, 1∶1∶4) to provide the title compound as a white solid (205 mg, 25%). **^1^H NMR** (400 MHz, DMSO-d_6_) δ 8.00–7.59 (m, 4H), 4.50 (t, *J* = 7.8 Hz, 2H), 4.04 (t, *J* = 7.8 Hz, 2H), 2.15 (s, 3H). **^13^C NMR**(101 MHz, DMSO-d_6_) δ 213.5, 167.3, 156.4, 151.4, 147.3, 134.5, 131.3, 130.5, 123.1, 113.8, 45.7, 37.3, 23.7. **LRMS** (ESI): *m/z*: 445 [M+H]^+^ (100%), 446 ([M+H]^+^ (100%).

#### *N*-3-(*N* ^2^-acetylguanin-7-yl)propylphthalimide (**19d**)

A mixture of *N* ^2^-acetyl-8-bromoguanine (**18**) (810 mg, 2.98 mmol), *N*-(3-bromopropyl)phthalimide (1.10 g, 4.03 mmol), DIPEA (1.60 mL, 9.00 mmol) in DMF (10 mL) was refluxed at 100°C overnight. The solvent was evaporated *in vacuo*, diluted with water (50 mL) and extracted with chloroform (3 × 50 mL). The pooled organic phases were dried over MgSO_4_, then evaporated *in vacuo*. The residue was purified by silica gel chromatography (MeOH/CHCl_3_, 1∶19) providing the title compound as a white solid (276 mg, 20%). **^1^H NMR** (400 MHz, DMSO-d_6_) δ 7.93–7.70 (m, 4H), 4.34 (t, *J* = 7.0 Hz, 2H), 3.63 (t, *J* = 7.0 Hz, 2H), 2.27–2.04 (m, 5H). **^13^C NMR** (101 MHz, DMSO-d_6_) δ 213.4, 167.8, 156.3, 151.4, 147.4, 134.3, 131.6, 130.1, 122.9, 113.5, 44.7, 34.6, 28.6, 23.6. **LRMS** (ESI): *m/z*: 459 [M+H]^+^ (100%), 460 [M+H]^+^ (100%).

#### 7-(2-Hydroxyethyl)-8-mercaptoguanine (21a)

To a solution of *N* ^2^-acetyl-8-bromo-7-(2-hydroxyethyl)guanine (**19a**) (10 mg, 0.03 mmol) in water (4 mL) and acetonitrile (2 mL) was added sodium thiosulfate (10 mg, 0.10 mmol) and aluminium chloride (0.02 mmol). The solution was refluxed for 24 h, then 1M HCl added and the solution stirred for a further 2 h. The solutionwas subjected to reverse phase chromatography (C18, isocratic 0.1% TFA in water) to isolate the title compound as a white powder (5 mg, 69%). **Mp**>300°C, **^1^H NMR** (400 MHz, DMSO-d_6_) δ 10.91 (s, 2H), 6.54 (s, 5H), 4.79 (t, *J* = 5.7 Hz, 3H), 4.25 (t, *J* = 6.7 Hz, 6H), 3.64 (dd, *J* = 6.7, 5.7 Hz, 6H).**^13^C NMR** (101 MHz, DMSO-d_6_) δ 164.1, 154.1, 151.4, 149.5, 105.3, 58.5, 46.4. **LRMS** (ESI): *m/z*: 228.1 [M+H]^+^ (100%), **HRMS** (ESI): observed *m/z:* 226.039 [M-H]^-^; calculated *m/z:* 226.0404 [M-H]^-^, **RP-HPLC**: t_R_4.14 min, >98%.

#### 2-(8-Mercaptoguanin-7-yl)acetic acid- (21b)

To a solution of methyl-8-bromo-(*N* ^2^-acetylguanin-7-yl)acetate (**19b**) (95 mg, 0.28 mmol) in water (4 mL) and acetonitrile (2 mL) was added sodium thiosulfate (200 mg, 1.10 mmol,) and aluminium chloride (0.02 mmol). The solution was refluxed for 2 days, filtered and resuspended in water/methanol/dioxane (2∶1∶4), and the pH of the solution was brought to 13 by adding 1 M NaOH. The solution was stirred at 50°C for 2 h, then subjected to reverse phase chromatography (C18, isocratic 0.1% TFA in water) to isolate the title compound as a white solid (15 mg, 20%). **Mp** 247–253°C (dec), **^1^H NMR** (400 MHz, DMSO-d_6_) δ 10.99 (s, 1H), 6.62 (s, 1H), 4.87 (s, 1H).**^13^C NMR** (101 MHz, DMSO-d_6_) δ 168.6, 165.0, 154.2, 151.4, 149.2, 105.0, 45.4. **LRMS** (ESI): *m/z*: 242 [M+H]^+^ (100%), **HRMS** (ESI): observed *m/z:* 242.0341 [M+H]^+^; calculated *m/z:* 242.0342 [M+H]^+^, **RP-HPLC**: t_R = _4.23 min, >95%.

#### 7-(2-Aminoethyl)-8-mercaptoguanine (21c)

To a solution of of *N*-2-(8-bromo-*N* ^2^-acetylguanin-*7*-yl)ethylphthalimide (**19c**) (160 mg, 0.36 mmol) in water (12 mL) and acetonitrile (8 mL) was added with sodium thiosulfate (447 mg, 1.80 mmol) and aluminium chloride (0.02 mmol). The reaction was refluxed for 2 days, solvent removed *in vacuo* and the residue resuspended in methanol (1 mL) and hydrazine hydrate (12 µL, 0.36 mmol). The solution was stirred for 15 h, the subjected to reverse phase chromatography (C18, isocratic: 0.1% TFA in water) to provide the title compound as a white solid (10 mg, 32%). **Mp** 280–287°C (*dec*), **^1^H NMR** (400 MHz, DMSO-d_6_) δ 8.22 (s, 1H), 7.85 (s, 2H), 6.78 (s, 2H), 4.41 (t, *J* = 6.1 Hz, 1H), 3.16 (t, *J* = 6.1 Hz, 1H).**^13^C NMR** (101 MHz, DMSO-d_6_) δ 164.6, 154.5, 151.7, 150.1, 105.1, 42.5, 38.5. **LRMS** (ESI): *m/z*: 227.1 [M+H]^+^ (100%), **HRMS** (ESI): observed *m/z:* 225.0564 [M-H]^-^; calculated *m/z:* 225.0564 [M-H]^-^,**RP-HPLC**: t_R = _2.9 min, >98% (gradient).

#### 7-(3-Aminopropyl)-8-mercaptoguanine (21d)

A suspension of *N*-3-(*N* ^2^-acetylguanin-7-yl)propylphthalimide (**19d**) (150 mg, 0.33 mmol) in water (12 mL) and acetonitrile (8 mL) was added sodium thiosulphate (400 mg, 1.60 mmol) and aluminium chloride (0.02 mmol). The reaction was refluxed for 2 days. After cooling the mixture was concentrated to dryness under reduced pressure. The reaction was refluxed for 2 days, solvent removed *in vacuo* and the residue resuspended in methanol (1 mL) and hydrazine hydrate (12 µL, 0.36 mmol). The reaction was stirred for 15 h at rt and the the mixture subjected to reverse phase chromatography (C18, isocratic: 0.1% TFA in water) to provide the title compound as a white solid (12 mg, 44%). **Mp** 239–243°C (*dec*.), **^1^H NMR** (400 MHz, D_2_O) δ 4.41 (t, *J* = 6.6 Hz, 2H), 3.08 (t, *J* = 6.6 Hz, 2H), 2.22 (t, *J* = 6.6 Hz, 2H). **^13^C NMR** (101 MHz, D_2_O) δ 163.0, 154.4, 153.0, 150.1, 106.0, 42.0, 36.3, 26.5. **LRMS** (ESI): *m/z*: 241.1 [M+H]^+^ (100%), **HRMS** (ESI): observed *m/z:* 241.0942 [M+H]^+^; calculated *m/z:* 241.0827 [M+H]^+^, **RP-HPLC**: t_R = _3.76 min, >98%.

#### 7-(2-Guanidinoethyl)-8-mercaptoguanine (**21e**)

A mixture of 7-(2-aminoethyl) 8-mercaptoguanine (**21c**) (10 mg, 0.04 mmol) and pyrazolecarboxamidine (7 mg, 0.10 mmol) in DMF was stirred at 50°C for 2 days. The resulting mixture was concentrated to dryness under reduced pressure. The crude product was purified using reverse phase chromatography (C18, isocratic: 0.1% TFA in water) to provide the title compound as a white solid (5.00 mg, 45%). **Mp** 256–262°C (*dec*), **^1^H NMR** (400 MHz, DMSO-d_6_) δ 11.18 (s, 1H), 7.64 (t, *J* = 6.2 Hz, 1H), 6.75 (s, 2H), 4.29 (t, *J* = 6.4 Hz, 2H), 3.47 (t, *J* = 6.4 Hz, 2H). **^13^C NMR** (101 MHz, DMSO-d_6_) δ 164.4, 156.9, 154.3, 151.5, 149.9, 104.8, 45.7, 42.8. **LRMS** (ESI): *m/z*: 269.1 [M+H]^+^ (100%), **HRMS** (ESI): observed *m/z:* 269.0936; calculated *m/z:* 269.0928 [M+H]^+^, **RP-HPLC**: t_R_4.23 min, >98%.

### Surface Plasmon Resonance (SPR)

All SPR binding experiments were performed as described previously [8]. The only difference was the use of a sulfhydryl reactive maleimide-activated biotin derivative (Thermo Scientific, 1-biotinamido-4-(4′-[maleimidoethylcyclohexane]-carboxamido)butane. The maleimide-activated biotin was attached to the exposed surface cysteine residue of *Sa*HPPK according to manufacturer’s instructions. The resulting site-specific biotinylated protein was immobilized onto the sensor chip surface using the Biotin capture kit (GE Healthcare). All analogues were serially diluted (either 2- or 3-fold from 126 µM down to 1.5 µM) in SPR binding buffer (50 mM HEPES, 150 mMNaCl, 1 mM TCEP, 0.05% (v/v) Tween-20, 10 mM MgCl_2_, 5% (v/v) DMSO, pH 8.0) and injected for 30 sec contact time at 60 µL/min and then allowed to dissociate for 60 sec. Binding sensorgrams were processed using the Scrubber (version 2c, BioLogic Software, Campbell, Australia). To determine the binding affinity (equilibrium dissociation constant; *K_D_*), responses at equilibrium for each compound were fit to a 1∶1 steady state affinity model available within Scrubber.

### Isothermal Titration Calorimetry (ITC)

Experiments were performed using an iTC200 instrument (MicroCal) at 298 K, with the ligands titrated into solutions of *Sa*HPPK using 18×2.2 µL injections. Data were fitted using Origin software to yield the thermodynamic parameters, Δ*H*, *KD* and N (the binding stoichiometry), assuming a cell volume of 0.2 mL. These were then used to calculate the Gibb’s free energy of binding, Δ*G* (-RT.ln*K*_a_), and entropy of binding, Δ*S* (using Δ*G* = Δ*H* - TΔ*S*). A stock solution of *Sa*HPPK was dialyzed overnight into 50 mM HEPES, 1 mM TCEP, 10 mM MgCl_2_, pH 8.0 buffer with the addition of 5% DMSO (v/v) prior to running the experiment. For titrations with compounds **21a**–**e**, *Sa*HPPK was typically at 30 µM and the ligand stocks were at 1**–**1.5 mM dissolved in the above buffer then diluted into more of the same buffer. There was no apparent issue with limited solubility of **21a–c** compromising either the stock solutions or the injected concentrations.

### X-ray Crystallization and Structure Determination

Crystallization experiments were performed as described previously [9]. Briefly, co-crystallization was set-up in the JCSG+ Suite commercial crystal screens (Qiagen) at 281 K using sitting-drop vapor-diffusion method with droplets consisting of 150 nL protein solution and 150 nL reservoir solution and a reservoir volume of 50 µL. Crystals of the *Sa*HPPK in complex with 7-(2-hydroxyethyl)guanine (**21a**) were observed in conditions containing 240 mM sodium malonate and 20% polyethylene glycol 3350. Data were collected at the MX-2 beamline of the Australian Synchrotron (see Table 3 for statistics) using a one degree oscillation angle, 360 frames were obtained for a complete data set. These data were indexed using XDS [38] and scaled using SCALA [39].

The *Sa*HPPK structure (3QBC) was used to solve the initial phases of the binary complex by molecular replacement using Phaser [40]. Refinement was performed using *REFMAC*5 [41] and the Fourier maps (2F_O_-F_C_ and F_O_-F_C_) were visualized in *Coot* [42]. After several rounds of manual rebuilding, **21a** and water molecules were added and the model further refined to a resolution of 1.85 Å (*R*_free_ (%) = 26.4, *R*_work_ (%) = 20.9).

The coordinates of *Sa*HPPK in complex with **21a** have been deposited at the Protein Data Bank with accession number 4ad6.

### NMR Spectroscopy

^15^N-labelled protein samples for NMR spectroscopy were prepared as described [8]. 2D soFast ^15^N HMQC [43] NMR experiments were recorded on a Varian Inova 600 MHz NMR spectrometer equipped with a cryoprobe and Z axis gradient on samples of ∼100 µM ^15^N-labelled *Sa*HPPK dissolved in 50 mM HEPES buffer (pH 8.0, 90% H_2_O 10% D_2_O, 1% sorbitol) by titrating in aliquots from a 25 mM stock of **21a** dissolved in DMSO-D_6_.

## Supporting Information

Figure S1 **SPR raw data (** ***top*** **) and steady-state response curves (** ***bottom*** **) for the binding of** ***C*** **^8^- (10a–f),** ***N*** **^9^-(15a–d) and** ***N*** **^7^-(21a–e) substituted analogues to** ***Sa*** **HPPK.**
(TIFF)

Figure S2 **SPR raw data (** ***top*** **) and steady-state response curves (** ***bottom*** **) for the binding of compounds 21a, 21c, 21d and 21e to** ***Sa*** **HPPK.**
(TIFF)

Figure S3 **ITC raw data (** ***top*** **) and integrated data (** ***bottom*** **) for the titration of SaHPPK with compounds 21a, 21c, 21d and 21e.**
(TIFF)
