# Supplementary figures and images for: Exploring the Chemical Space around 8-Mercaptoguanine as a Route to New Inhibitors of the Folate Biosynthesis Enzyme HPPK
Source: PLoS One. 2013 Apr 2;8(4):e59535. doi: 10.1371/journal.pone.0059535 (PMC3614987; doi:10.1371/journal.pone.0059535)

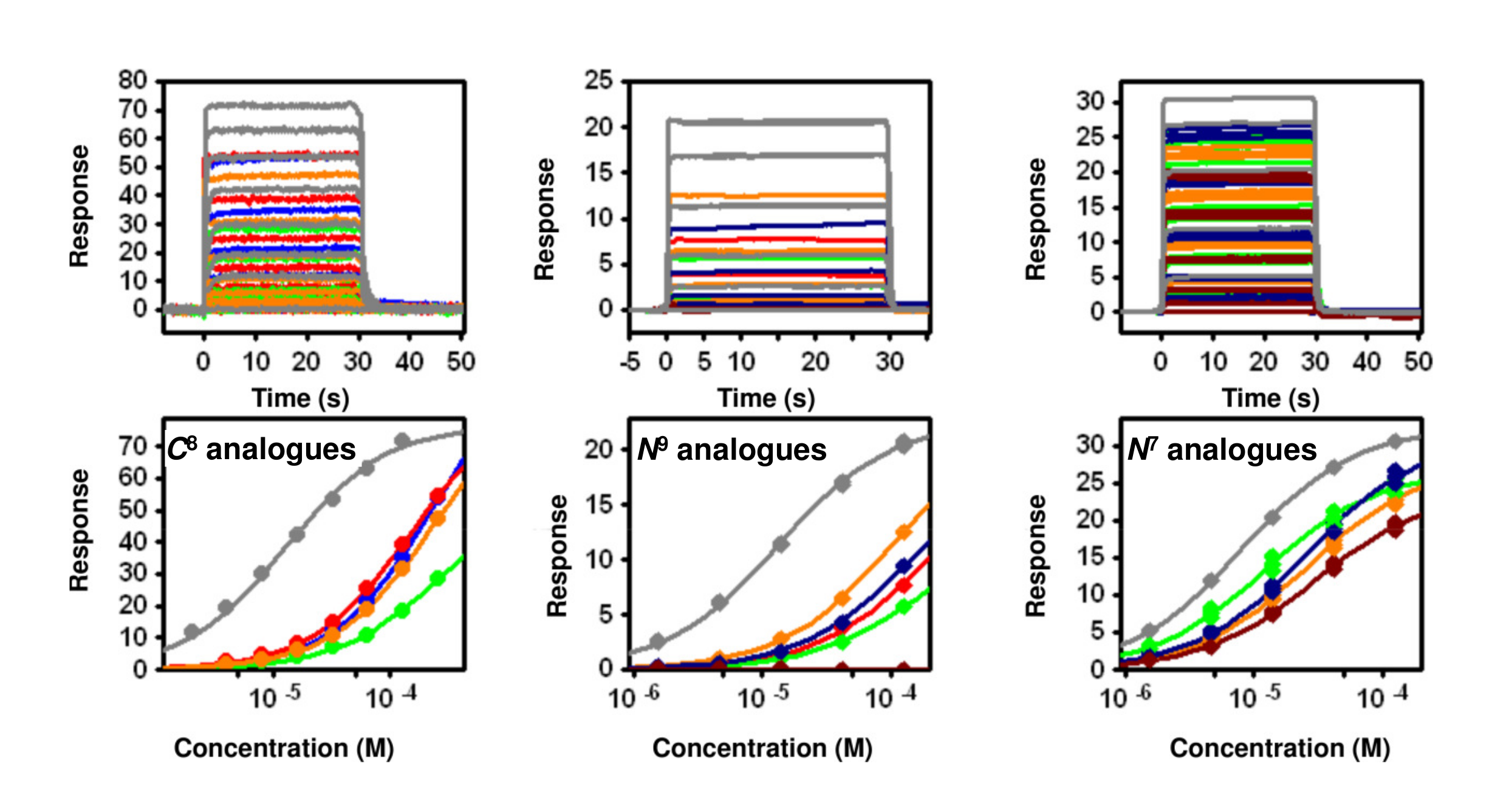

Supplement: Figure S1 — SPR raw data ( top ) and steady-state response curves ( bottom ) for the binding of C 8- (10a–f), N 9-(15a–d) and N 7-(21a–e) substituted analogues to Sa HPPK. (TIFF) [file pone.0059535.s001.tiff]

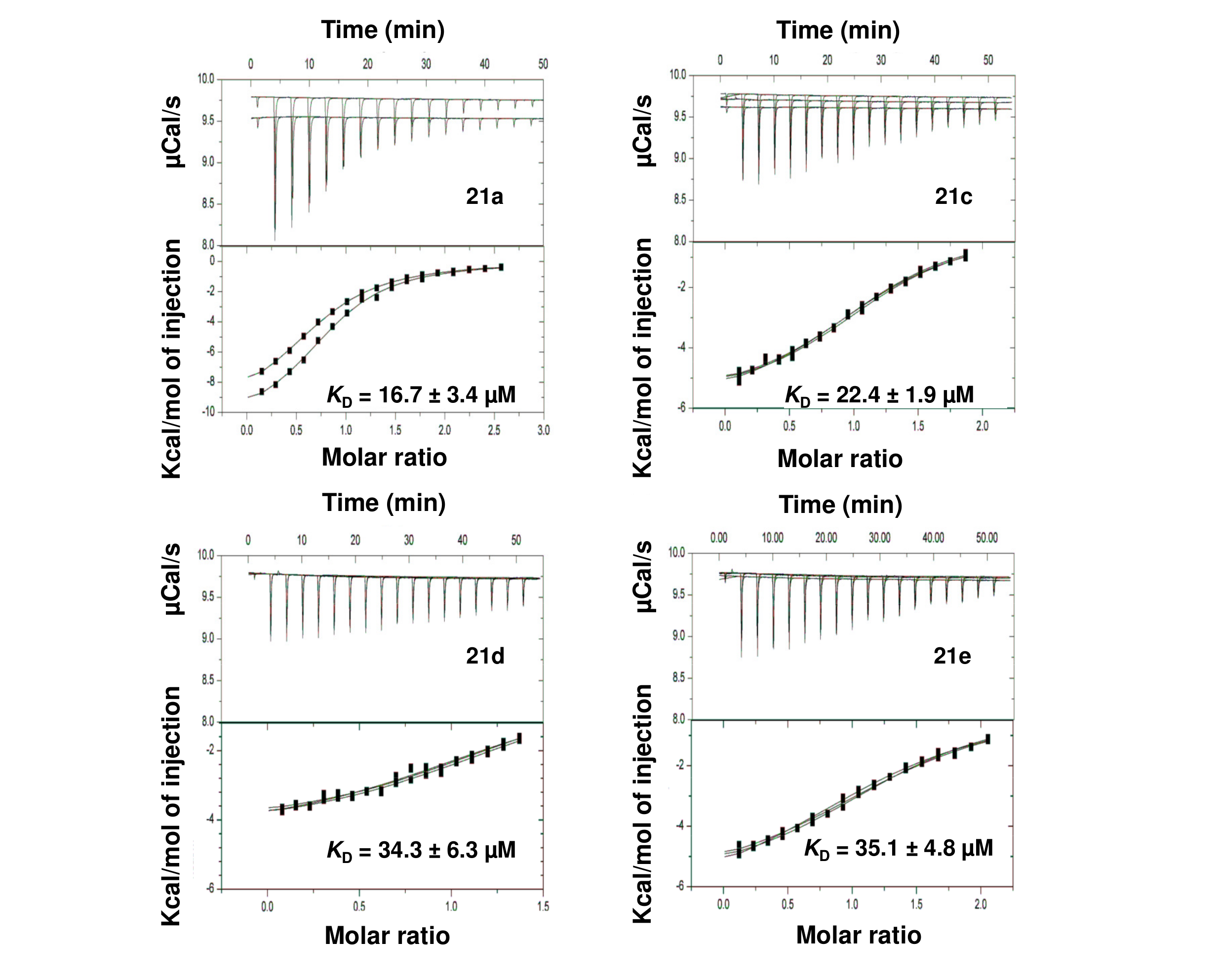

Supplement: Figure S3 — ITC raw data ( top ) and integrated data ( bottom ) for the titration of SaHPPK with compounds 21a, 21c, 21d and 21e. (TIFF) [file pone.0059535.s003.tiff]
